# Nerve Conduction and F-wave Findings in Patients With Postoperative Numbness After Reverse Shoulder Arthroplasty: A Case Series

**DOI:** 10.7759/cureus.106811

**Published:** 2026-04-10

**Authors:** Shotaro Teruya, Kazuhiro Ikeda, Hiroki Yamada, Hiromitsu Tsuge, Akira Ikumi, Shinzo Onishi

**Affiliations:** 1 Department of Orthopedic Surgery, Institute of Medicine, University of Tsukuba, Tsukuba, JPN; 2 Department of Orthopedic Sports Medicine, Institute of Health and Sport Sciences, University of Tsukuba, Tsukuba, JPN

**Keywords:** cubital tunnel syndrome, f-wave, nerve conduction studies (ncs), postoperative numbness, reverse shoulder arthroplasty (rsa)

## Abstract

Background: Neurologic symptoms such as ulnar-sided numbness can occur after reverse shoulder arthroplasty (RSA), and the lesion level may not be uniform across patients. This case series aimed to describe postoperative numbness and electrophysiological findings, with an exploratory assessment of F-wave findings in addition to standard nerve conduction studies.

Methods: We retrospectively analyzed patients who underwent RSA and had both preoperative and postoperative nerve conduction studies. Postoperative numbness was assessed clinically at six weeks. Motor and sensory conduction studies (MCS/SCS) in the ulnar nerve distribution and F-wave parameters were evaluated. F-wave abnormality was defined as either 1) prolongation of the minimum F-wave latency with a pre-to-post change of ≥2.0 ms or 2) reduced F-wave persistence of 50% or less.

Results: Thirteen patients were included. Postoperative numbness was observed in four patients (30.8%). Overall, five patients (38.5%) met the criteria for F-wave abnormality. F-wave abnormalities were observed in three of four patients with postoperative numbness and in two of nine patients without numbness. Two patients met electrodiagnostic criteria for cubital tunnel syndrome, and both had postoperative numbness. Additionally, two patients demonstrated isolated F-wave abnormalities with normal MCS/SCS, which may indicate electrophysiological changes not captured by conventional distal conduction studies alone.

Conclusions: In this case series of RSA patients, postoperative ulnar-sided numbness showed heterogeneous electrophysiological patterns. F-wave assessment, combined with MCS/SCS, may provide supplementary electrophysiological information in cases with normal distal conduction findings. Larger studies with longitudinal follow-up are warranted to clarify symptom-electrophysiology relationships and clinical implications.

## Introduction

Neurologic complications after reverse shoulder arthroplasty (RSA) are important adverse events, with reported incidence ranging from 1.3% to 2.4% [1-3]. In RSA, a glenosphere (hemispherical implant) is placed on the glenoid side, and the humeral head is resected with insertion of a flat implant on the humeral side, thereby distalizing the humerus and increasing deltoid tension [4]. Owing to these procedure-specific characteristics, neurologic injury is thought to occur when the brachial plexus is placed under traction due to humeral distalization and/or excessive intraoperative traction maneuvers [5-7].

Although previous reports have examined the incidence and clinical manifestations of neurologic injury after RSA, few studies have objectively evaluated the anatomical level of the lesion. In particular, mild neurologic injury without abnormalities on motor and sensory nerve conduction studies (MCS/SCS), as well as proximal lesions at the brachial plexus level, can be difficult to detect using conventional nerve conduction testing alone.

The F-wave is a compound action potential elicited by electrical stimulation of a motor nerve, generated by recurrent discharge of anterior horn cells, and has been considered useful for evaluating proximal lesions of peripheral nerves [8]. Notably, F-wave parameters may provide supplementary electrophysiological information beyond conventional distal conduction studies. Therefore, we described postoperative ulnar-sided numbness and electrophysiological findings after RSA, with exploratory assessment of F-wave findings in addition to standard MCS/SCS on a case-by-case basis.

## Materials and methods

Study design and participants

**Figure 1:**
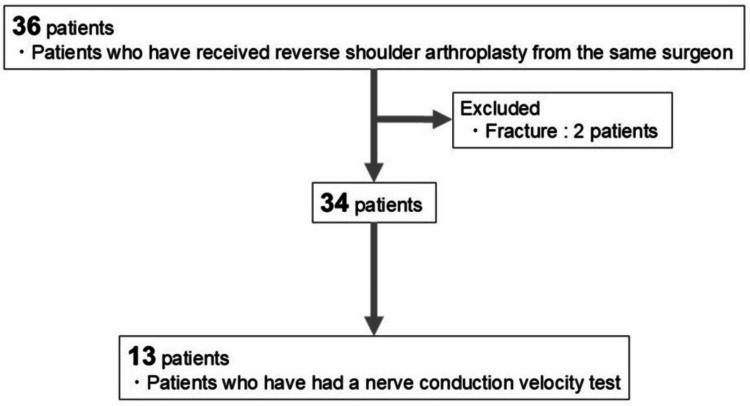
Patient selection flow diagram Patients who underwent reverse shoulder arthroplasty between April 2019 and April 2020 were screened. Fracture cases and those without available pre- and postoperative nerve conduction studies were excluded, leaving 13 patients for analysis

This study was a retrospective case series based on medical records (level IV evidence). Institutional Review Board approval was obtained (approval number: R07-290). Among 36 patients who underwent RSA at our institution between April 2019 and April 2020, fracture cases were excluded, and 13 patients who were able to undergo nerve conduction studies both preoperatively and postoperatively were included (Figure 1). All procedures were performed using the Aequalis Reversed Shoulder System (Stryker, Kalamazoo, MI). Postoperative numbness was defined as patient-reported paresthesia and/or hypoesthesia in the ulnar distribution (little finger, ulnar half of the ring finger, and/or ulnar forearm), assessed at six weeks postoperatively by the treating surgeon. No patients reported similar symptoms preoperatively.

Nerve conduction studies

Nerve conduction studies were performed preoperatively and at six weeks postoperatively. All examinations were performed in the same electrodiagnostic examination room under routine room conditions. Patients were examined in the supine position, with the shoulder abducted to 90°, the elbow flexed to 90°, and the forearm in the neutral position. The electrode placement, stimulation sites, and outcome measures for the MCS, SCS, and F-wave study are summarized in Table 1.

**Table 1:** Nerve conduction study protocol and recording conditions ^*^F-wave persistence was calculated from 16 consecutive stimulations

Parameter	Motor conduction study	Sensory conduction study	F-wave study
Recording electrode	Belly of the abductor digiti minimi muscle	Metacarpophalangeal joint of the little finger	Belly of the abductor digiti minimi muscle
Reference electrode	Metacarpophalangeal joint of the little finger	Proximal interphalangeal joint of the little finger	Metacarpophalangeal joint of the little finger
Stimulation site(s)	Wrist (70 mm proximal to the recording electrode), 40 mm distal to the ulnar groove, and 60 mm proximal to the ulnar groove	Wrist (100 mm proximal to the recording electrode), 40 mm distal to the ulnar groove, and 60 mm proximal to the ulnar groove	60 mm proximal to the ulnar groove
Outcome measure(s)	Motor nerve conduction velocity	Sensory nerve conduction velocity	Minimum F-wave latency and F-wave persistence^*^

Definitions of neurologic impairment

Cubital tunnel syndrome was defined as meeting at least one of the following electrodiagnostic criteria [9]: a motor nerve conduction velocity (MCV) across the above-elbow to below-elbow (AE-BE) segment of less than 50 m/s, an MCV across the AE-BE segment more than 10 m/s slower than that across the below-elbow to wrist segment on the same side, or a reduction of 20% or more in compound muscle action potential amplitude with above-elbow stimulation compared with below-elbow stimulation. Isolated F-wave abnormality was defined as normal MCS/SCS findings with abnormal F-wave parameters only. F-wave abnormality was defined as either prolongation of the minimum F-wave latency, with an increase of 2.0 ms or more from the preoperative value, or reduced F-wave persistence of 50% or less [10,11]. The threshold for minimum F-wave latency prolongation was selected to exceed the range of measurement variability reported in normative studies and to reflect a clinically meaningful change at the individual level, given that the preoperative value was used as a personal baseline. By using the preoperative value as an individual baseline, we aimed to minimize the influence of interindividual variability in F-wave parameters and to capture postoperative changes at the individual level.

## Results

Baseline characteristics of the 13 included patients are summarized in Table 2. The mean age was 69.9 ± 11.6 years; there were two men and 11 women. In the no-numbness group, the underlying diagnoses were cuff tear arthropathy in five patients, rheumatoid arthritis in two patients, and massive rotator cuff tears in two patients. In the numbness group, the indications were revision arthroplasty (conversion RSA) in two patients and massive rotator cuff tears in two patients (Table 2).

**Table 2:** Baseline characteristics of the 13 included patients SD: standard deviation; BMI: body mass index; RSA: reverse shoulder arthroplasty

Characteristic	Value
Age (years, mean ± SD)	69.9±11.6
Sex	
Male	2
Female	11
Height (cm, mean ± SD)	149.8 ± 7.9
BMI (kg/m², mean ± SD)	21.9 ± 3.1
Underlying diagnosis	
Cuff tear arthropathy	5
Rheumatoid arthritis	2
Massive rotator cuff tear	4
Conversion RSA	2
Postoperative numbness	4

Neurologic symptoms

Postoperative numbness was observed in four patients (30.8%). The predominant symptoms were paresthesia on the ulnar side of the hand and hypoesthesia along the ulnar aspect of the forearm. No patient demonstrated overt motor paralysis.

F-wave findings

**Figure 2:**
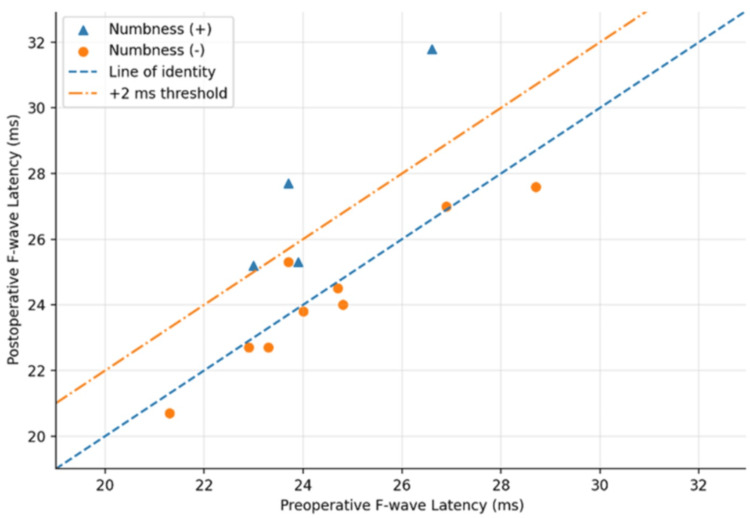
Preoperative vs. postoperative F-wave minimum latency Scatter plot comparing preoperative and postoperative F-wave minimum latency for each case. The dashed line indicates the line of identity (postoperative = preoperative). The dash-dotted line indicates the threshold for clinically meaningful prolongation (postoperative = preoperative + 2.0 ms). Triangles represent patients with postoperative numbness, and circles represent those without postoperative numbness. Clinically meaningful prolongation was defined as ΔF-wave minimum latency ≥2.0 ms from the preoperative value

Preoperative and postoperative minimum F-wave latency and F-wave persistence are summarized in Table 3. The mean preoperative minimum F-wave latency was 24.4 ± 2.0 ms, and the mean postoperative value was 25.3 ± 2.8 ms. Clinically meaningful prolongation, defined as an increase of 2.0 ms or more from the preoperative value, was observed in three patients (23.1%) (Figure 2). All three patients were in the numbness group: case 1 showed a change from 23.7 to 27.7 ms, case 2 from 26.6 to 31.8 ms, and case 3 from 23.0 to 25.2 ms (Table 3, Figure 2).

**Table 3:** Individual patient characteristics and F-wave parameters CuTS: cubital tunnel syndrome ^*^Clinically meaningful prolongation was defined as ΔF-wave minimum latency ≥2.0 ms from the preoperative value ^**^F-wave persistence was defined as ≤50% at postoperative assessment [11] and was calculated from 16 consecutive stimulations

Case	Postoperative numbness	Height (cm)	Weight (kg)	CuTS	Preoperative F-wave		Postoperative F-wave	
					Latency (ms)	Persistence (%)	Latency (ms)	Persistence (%)
1	+	148	53	-	23.7	87	27.7^*^	50^**^
2	+	168.5	55	-	26.6	100	31.8^*^	50^**^
3	+	148.4	36.3	+	23	93	25.2^*^	100
4	+	151.3	62.6	+	23.9	87	25.3	100
5	-	150.4	54.2	-	21.3	81	20.7	12^**^
6	-	140.5	41	-	24.8	87	24	81
7	-	154.8	51.6	-	26.9	81	27	62
8	-	147.5	42.8	-	24	75	23.8	100
9	-	144.5	39.1	-	23.7	68	25.3	87
10	-	159	64.8	-	28.7	75	27.6	50^**^
11	-	142	39	-	23.3	81	22.7	75
12	-	152.6	54.5	-	24.7	93	24.5	87
13	-	142	47.6	-	22.9	81	22.7	81

Neurologic classification

Two patients (15.4%) met the electrodiagnostic criteria for cubital tunnel syndrome, and both reported postoperative numbness. In addition, two patients (15.4%) showed isolated F-wave abnormalities with normal MCS and SCS findings, which may indicate electrophysiological changes not captured by conventional distal conduction studies alone. When stratified by postoperative numbness status, F-wave abnormalities were observed in three of four patients in the numbness group and in two of nine patients in the no-numbness group (Table 3).

## Discussion

The primary implication of this study is that even when patients present with similar ulnar-sided numbness, the electrophysiological pattern may not be uniform across cases. While some patients met the electrodiagnostic criteria for cubital tunnel syndrome, others showed abnormal F-wave findings despite normal MCS and SCS, which may indicate electrophysiological changes not captured by conventional distal conduction studies alone. However, F-wave abnormalities alone are insufficient to confirm lesion localization, and findings should be interpreted in conjunction with clinical and other electrophysiological data.

Mechanical stress on the brachial plexus related to humeral distalization during RSA has been suggested [12], and intraoperative traction maneuvers and procedure-related factors may influence postoperative neurologic impairment [13]. In a clinical study of reverse total shoulder arthroplasty, postoperative neurologic deficits were reported to correlate with the degree of humeral distalization, supporting traction-related mechanisms in at least a subset of patients [14].

Moreover, a case series describing severe peripheral nerve injuries following reverse total shoulder arthroplasty reported variable injury patterns, highlighting that neurologic impairment after RSA may not be uniform across patients [15]. These reports are consistent with our observation that mild subjective symptoms may coexist with electrophysiological changes.

From a clinical perspective, when ulnar-sided numbness occurs after RSA, it is important to consider that some patients may demonstrate abnormal F-wave parameters despite preserved distal conduction. Thus, even when symptoms appear similar, heterogeneous mechanisms may be present, and interpretation based on multiple indicators (clinical findings, MCS/SCS, and F-wave parameters) may provide a more comprehensive assessment than distal conduction findings alone. A particular strength of the present study is the availability of paired preoperative and postoperative measurements, which allowed F-wave changes to be assessed at the individual level rather than relying solely on population-based normative references. This individual-baseline approach may help reduce the influence of interindividual variability and enhance sensitivity to postoperative changes.

This study has several limitations. First, the sample size was limited, and the assessment was centered on the six-week postoperative time point, which may not fully capture longitudinal neurologic changes. In addition, the small sample size limits interpretation of the descriptive distribution of F-wave abnormalities according to postoperative numbness status, and these patterns should not be interpreted as evidence of an association. Second, F-wave parameters can be influenced by measurement conditions and interindividual variability [16], and the thresholds used to define F-wave abnormality were adopted from existing references and applied exploratorily in this RSA cohort; their validity in this setting has not been established. Needle electromyography and control nerve testing were not performed; therefore, isolated F-wave abnormalities should be interpreted cautiously. Finally, two patients in the numbness group had undergone conversion RSA, which may carry a different risk profile from primary RSA, thereby limiting generalizability across subgroups.

## Conclusions

In this case series, nerve conduction studies in patients who developed ulnar-sided numbness after RSA suggested heterogeneous electrophysiological patterns even among patients with similar symptoms. Specifically, while some patients met electrodiagnostic criteria for cubital tunnel syndrome, others exhibited isolated F-wave abnormalities with normal MCS/SCS findings, which may indicate electrophysiological changes not captured by distal conduction studies alone. Using the present operational definitions of F-wave abnormality (ΔFmin ≥ 2.0 ms and F-wave persistence ≤ 50%) may help characterize cases in which distal conduction findings are preserved. When evaluating postoperative ulnar-sided numbness after RSA, clinicians should interpret clinical and electrophysiological findings in an integrated manner. Further studies with larger cohorts and longitudinal follow-up are warranted to clarify the relationship between symptom course and electrophysiological findings.
